# Laser Thermal Wave Diagnostics of the Thermal Resistance of Soldered and Bonded Joints in Semiconductor Structures

**DOI:** 10.3390/s23073590

**Published:** 2023-03-30

**Authors:** Alexey Glazov, Kyrill Muratikov

**Affiliations:** Ioffe Institute, Saint-Petersburg 194021, Russia

**Keywords:** photothermal technique, mirage-effect, thermal waves, interfacial thermal resistance, thermal impedance

## Abstract

This paper is a review of recent applications of a laser photothermal mirage technique for sensing and measuring the thermal resistance of joint layers in modern electronic devices. A straightforward theoretical model of the interfacial thermal resistance based on the formation of a thin intermediate layer between jointed solids is described. It was experimentally shown that thermal properties of solder layers cannot be evaluated simply on the base of averaging the thermal properties of solder components. The review presents the laser thermal wave methodology for measuring thermal parameters of soldered and adhesively bonded joints. The developed theoretical model makes it possible to carry out a quantitative estimation of local thermal conductivities of joints and their thermal resistances by fitting theoretical results with experimental data obtained by the laser beam deflection method. The joints made with lead-containing and lead-free solders were studied. The anomalous distribution of thermal properties in the solder layer is explained by the diffusion of various atoms detected by energy dispersive X-ray spectroscopy. The laser beam deflection method made it possible to reveal a strong influence of the surface pretreatment quality on the interfacial thermal resistance.

## 1. Introduction

Semiconductor electronic components have been a growing global market in the past decades [1]. The successful growth of the electronic sector of the economy is essentially determined by the quality of certain technological operations. Along with the process of implementation of electronic devices, an important stage is their assembly into the final product. Such technological operations as soldering [2] and bonding [3] play an important role at this stage of manufacturing. Due to their high electrical, thermal, and mechanical properties, solders are the most widely used materials for joining in assembling an electronic product. The characteristics of the solder joints are very important because the failure of an entire electronic assembly is often caused by the failure of solders. Certain high-power electronic devices operate at elevated temperatures, and the use of solder joints is undesirable. In such cases, bonding is often used instead of soldering. The bonding also provides electronic components with a high level of quality and performance, allowing electronic devices to operate at higher temperatures compared to soldering. A significant reduction in sizes of electronic components and the corresponding increase in their density due to technological progress in semiconductor device production have led to a significant increase of thermal energy flowing through their interfaces. The lifetime of such electronic devices is usually determined by the exponential temperature dependence [4]. Therefore, the miniaturization of electronic devices has introduced an urgent need for a significant increase in the reliability of solder joints. Understanding the details of heat diffusion through interfaces in semiconductor devices is an urgent prerequisite to improving their productivity and reliability. Designing a thermomechanically reliable microelectronic assembly is a laborious and expensive process. Therefore, modeling and diagnostic methods have become useful tools to predict and control the thermomechanical reliability of solder joints.

A viscoplastic constitutive model, the Anand model [5], is commonly used to estimate the thermomechanical properties of metallic solders. Its constitutive equation takes into account the strain rate sensitivity, strain hardening, dynamic recovery, and temperature sensitivity of a metal solder. This model has been proven to correctly determine the solder behavior [2]. The mechanical parameters of the solders included in the model can be determined using the corresponding constitutive equations [6,7] or special mechanical tests. Temperature plays an important role in the analysis of thermomechanical processes within the framework of the Anand model. This model assumes that the temperature is known, while in practice it needs to be measured. Modern solders and bonding materials have acceptable mechanical, thermal, and electric properties. Their bulk mechanical and thermal properties are thoroughly studied, but their properties at interfaces and in thin joints have not been.

Usually, only average values of bulk thermal parameters are available for solders of different compositions [8,9]. However, heat transfer through contacts depends not only on the thermal conductivity of the solder, but also on a number of properties of the contacting surfaces, including thermal and acoustic parameters of joining materials, quality of mechanical and chemical treatment of surfaces, and the contact quality. In order to characterize the thermal quality of a joint, it is desirable to know not only its average thermophysical parameters, but also their local values. To take into account these factors, the heat diffusion through solid boundaries is usually characterized by Kapitza or thermal resistance (TR) [10,11,12]. It seriously affects the heat transfer through contacts of different materials in multilayer structures. Many microelectronic applications that are sensitive to heat dissipation require a low TR at interfaces. The kind of joining, the thermal conductivity of the joint material, and the pretreatment of the surfaces are of paramount importance for reducing the TR. The review focuses on the experimental measurement of the joint TR responsible for thermal barriers due to joint layer properties. The local deterioration of heat removal from the contact can lead to its overheating, the occurrence of thermal stresses and, ultimately, to the destruction of the joint. D. Straubinger et al. [13] demonstrated the reduction of shear strength in solder joints under current loading and isothermal aging. The formation of voids in the solder layer during the flow of current can also lead to the appearance and increase in the joint TR [14]. The voids are formed as a result of the electromigration of solder atoms when an electric current flows through the contact. The void formation is especially dangerous in semiconductor devices with high current densities. Such devices include, for example, photovoltaic solar cells with radiation concentrators working at high levels of light irradiation [15] and high-voltage pulse semiconductor switches based on a diode stack assembly [16]. A detailed theoretical and experimental consideration of electromigration of atoms and the dynamics of the void formation in solder layers was performed by F. Jia et al. [14].

To ensure the reliable operation of microelectronic devices, it is necessary to form perfect thermal contacts between the semiconductor substrate of the device and the heat sink. In such devices, ceramic substrates with high thermal conductivity are often used as a heat sink. Currently, the semiconductor part of the devices and heat sink ceramics are joined by soldering. For over fifty years, solder joints have been made primarily with two-component tin–lead solders. However, due to legal restrictions on the use of toxic substances, the use of lead was banned in the EU and later in other countries. Nowadays, much attention is paid to lead-free solder alloys instead of conventional Sn–Pb solders. Mechanical and thermal properties of various lead-free binary [17,18,19] and ternary solders [8,20,21] are actively investigated. Such solders demonstrate acceptable mechanical and thermal properties. At the same time, the following comments should be made regarding the situation with the use of lead-free solders in microelectronics. The most widely used lead-free solder alloy Sn-3.0Ag-0.5Cu has a relatively low melting point (217 °C), which is nevertheless noticeably higher than the melting point of eutectic Sn–Pb solder (183 °C) or Sn62/Pb36/Ag2 (179 °C) [22]. This difference can be significant when soldering semiconductor structures with thin layers (for example, quantum wells), since it can lead to additional undesirable atomic diffusion.

It should also be pointed out that lead-free solders tend to form tin whiskers, which can cause a short circuit [23]. The attempts to replace lead with other metals (such as Ag, Bi, Ni, and Au) have not yet been a success. In [23], it was shown that polyhedral oligomeric silsesquioxane (POSS)–silanol inhibits the whisker formation, and it can therefore be considered as a promising modifying material that enhances the reliability of solder joints. However, there are still important unresolved issues that do not allow the wide use of these new materials in industry. Issues such as strength, corrosion resistance and durability, cracking, and electromigration of atoms should be investigated in more detail for lead-free solders [24,25]. For these reasons, lead-containing solders are still used in heavy-duty electronic devices [26].

The TR of solid–solid contacts depends on many physical, thermal, mechanical, and geometrical parameters of contacting objects [27,28,29,30]. For dielectrics, the heat transfer is carried out by phonons [31], in metals by electrons [32], and in semiconductors by both phonons and electrons [33]. The contact TR is determined by complex processes of reflection and scattering of electrons and phonons on the contact surface, as well as by the possible electron–phonon interaction. For the dielectric–metal contact, depending on the direction of the heat flow, the contact TR is determined by transforming the heat flux of electrons (phonons) into the heat flux of phonons (electrons) [34]. If there are additional layers between the contacting objects, then the resulting TR also depends on parameters of these layers. Taking into account the spectral properties of phonons and the distributions of electrons in contacting materials, the calculation of the interfacial TR is a complex problem and is the subject of special studies [35]. A detailed calculation of the TR is beyond the scope of this review, and a phenomenological approach will be used for the interpretation of the obtained experimental data. The article presents and discusses the results of the application of the laser thermal wave methodology for the sensing and measurement of thermal parameters of soldered and adhesively bonded joints.

## 2. Experimental Techniques

Temperature measurements in microelectronics are often complicated by the fact that they must be performed on a micrometer or nanometer scale and with high temporal resolution [36]. At present, various techniques are used to characterize thermal transfer through the interfaces of solid structures. Some of them, e.g., 2ω [37] or 3ω [38,39] methods, are contact and require special preparation of samples for measurements. Laser-thermal-wave or photothermal techniques have long been used to study the thermal characteristics of inhomogeneous opaque objects. This also applies to interfaces between parts made of different materials. These methods provide the non-contact control of the diffusion of thermal waves excited by laser radiation. Due to the rapid decay of thermal waves with the length determined by the frequency of modulation of the heating, it is possible to implement the scanning microscopy mode with the submicron spatial resolution. The detection of temperature waves is possible in various ways. It is known that the methods that allow detecting only the integral surface temperature, for example, gas-cell microphone photoacoustics, are weakly sensitive to the presence of vertical thermal barriers. In this case, the local non-contact temperature detection is provided by such optical methods as infrared photothermal radiometry [40,41], laser thermoreflectance [42,43,44,45], photodeflection [46], and photothermal beam-deflection (PBD) [47,48,49] techniques. Briefly, these methods are as follows:

- Infrared photothermal radiometry—measurement of thermal radiation from the surface using a high-speed IR camera;
- Laser thermoreflectance technique—measurement of the reflectance of a test laser beam reflected from a surface at a heating point;
- Photodeflection technique—measurement of the deflection angle of the test laser beam reflected from the surface near the heating point, due to the thermoelastic change in the surface profile;
- Photothermal beam-deflection technique—measurement of the deflection angle of the test laser beam passing parallel to the surface through a thermal lens generated by the heat diffusion near the pump laser beam spot.

The advantages and disadvantages of these methods have been compared many times and are well-known, see, e.g., Ref. [50]. A significant advantage of the PBD technique in studying the properties and structure of soldered joints is a weak dependence on the optical quality of the surface. The probe beam does not interact with it, and it is independent from the thermoelastic and emission properties of materials. The disadvantage of the PBD technique is the lower spatial resolution since it is necessary to provide sufficient thermal wavelength in the gas to deflect the probe beam. The modulation frequency of the heating radiation does not exceed 10 kHz, which corresponds to a resolution of tens of microns. However, this is sufficient and, in addition, allows probing the connecting layer to a greater depth. For this reason, in many cases, the PBD method has proven to be more appropriate for the thermal evaluation of soldered devices [50] with interfaces perpendicular to the surface. Other methods mentioned are widely used for studying the thermal properties of thin films and thermal barriers when the layers are parallel to the surface.

**Figure 1:**
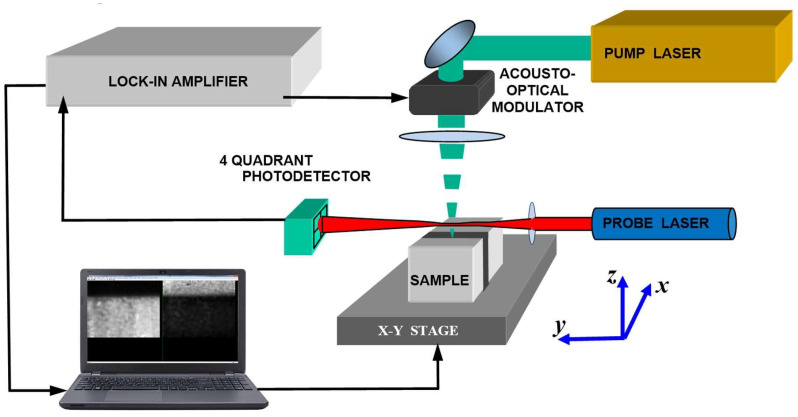
General scheme of the laser thermal wave microscope with the probe-beam deflection sensing.

The common block scheme of the PBD experimental setup is presented in Figure 1. When the thermal waves are generated by the focused radiation of an Ar–ion laser or a solid-state laser operating at the wavelengths of 0.514 and 0.532 μm, correspondingly, the heat source can be considered as a surface one. The intensity of the pump laser radiation was modulated in time by an acousto-optic modulator in the range from 1 kHz to 10 kHz. The radiation intensity was small enough and made no changes in the objects under investigation. The radiation of a low-pointing-noise He–Ne laser serves as a probe beam. The spot size of the pump laser on the sample was no more than 5 micron for most experiments presented below. The minimum probe beam waist was about 25 micron. There are two possible modes of the PBD signal detection: normal and transverse, in accordance with the direction perpendicular or parallel to the surface, in which the probe-beam deflection is detected by a position-sensitive photodetector. In this review, we focused only on the normal PBD signal. A lock-in amplifier was used for measuring the output of the photodetector. Two-dimensional PBD images of the sample areas near the joint layer were formed by moving the sample in two directions by step motors. The PBD images were used to exclude from consideration the near surface areas with defects such as Kirkendal voids [51]. These subsurface cavities are invisible through an optical microscope, but they are well-identified by the photothermal microscopy. Cross sections of the photothermal images without defects, perpendicular to the plane of soldered contacts, were used for the analysis of thermal waves. 

## 3. Theoretical Model for Evaluation of Thermal Properties of Multilayer Objects with Real and Reactive Thermal Interface Resistances

The problem of nonstationary thermal diffusion in two or more layer structures in the presence of the interfacial TR has been considered by many authors [52,53,54,55,56]. Usually, the TR of a contact at *x* = 0 is determined by the relationship

(1) $$\Delta T\left(t\right)=R_{th}\dot{Q}\left(t\right),$$

where ΔT is the temperature difference between contacting objects, $R_{th}$ is the total TR of the contact, and $\dot{Q}\left(t\right)$ is the rate of the steady heat transfer between the two surfaces.

In the form presented, the TR is a characteristic of the contact. When studying the properties of contacting materials, it is more convenient to use the concept of the specific TR defined by the relation

(2) $$\Delta T\left(t\right)=r_{th}\dot{q}\left(t\right),$$

where $r_{th}=R_{th}A$ is the TR per unit area of a piece of material or the specific TR, $\dot{q}=\dot{Q}/A$ is the heat flux density through the contact, and *A* is the contact area.

Further in the article, the specific TR will be used since it is a characteristic of materials, rather than that of the contact area. The Fourier law relates the heat flux density to the temperature gradient by the relation

(3) $$\dot{q}=-K\frac{\partial T}{\partial x},$$

where *K* is the thermal conductivity of the material.

Equations (2) and (3) make it possible to relate the TR of the contact to the behavior of the temperature near it

(4) $$\Delta T\left(t\right)=-r_{th}K{\left.\frac{\partial T}{\partial x}\right|}_{x=0},$$

**Figure 2:**
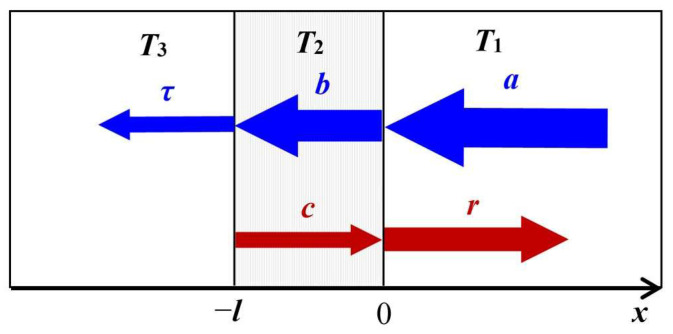
Geometry of propagation of the plane thermal waves in a three-layer object.

In the case of a constant heat flux, the TR depends only on the thermal properties of the intermediate layer between the contacting materials. In the case of unsteady heat fluxes, the TR of the contact may depend on the frequency. This situation can be illustrated by the example of the simplest structure shown in Figure 2. Using the concept of incident and reflected thermal waves and following the approach developed in [57,58], the alternative temperature components for three regions can be presented in the form of plane waves

(5) $$T_{1}\left(x,t\right)=(ae^{-iq_{1}x}+re^{iq_{1}x})e^{i\omega t}={\tilde{T}}_{1}\left(x,\omega \right)e^{i\omega t},\;x>0$$

(6) $$T_{2}\left(x,t\right)=(be^{-iq_{2}x}+ce^{iq_{2}x})e^{i\omega t}={\tilde{T}}_{2}\left(x,\omega \right)e^{i\omega t},\;-l<x<0$$

(7) $$T_{3}\left(x,t\right)=\tau e^{-iq_{3}x}e^{i\omega t}={\tilde{T}}_{3}\left(x,\omega \right)e^{i\omega t},\;x<-l$$

where *x* is the coordinate shown in Figure 2, *t* is the time, *l* is the thickness of the second layer, *q_n_* were obtained from the heat equations for each layer and *q_n_* = (−1 + *i*) (*ω*/2*κ_n_*)^1/2^, *ω* is the cyclic frequency of thermal waves, *κ_n_* is the thermal diffusivity of the *n*th layer, *a* is the amplitude of the incident thermal wave, and *r*, *b*, *c*, *τ* are coefficients to be determined from boundary conditions.

In [57,58], the coefficients at the exponents *r*, *c*, *d*, and *τ* as functions of the wave amplitude *a* were found in the analytical form by solving the heat equations for the structure shown in Figure 2 with common boundary conditions representing the continuity of temperature and heat flux at interfaces. In these publications, it was shown that in the case of a thin intermediate layer, the thermal problem for the structure in Figure 2 can be reduced to the problem of two regions between which there is the real TR *r_th_* and the reactive thermal conductance of the capacitive type *z_th_*

(8) $$T_{3}\left(0,\omega \right)-T_{1}\left(0,\omega \right)=-r_{th}K_{1}{\left.\frac{\partial T_{1}\left(x,\omega \right)}{\partial x}\right|}_{x=0},$$

(9) $${\left.K_{3}\frac{\partial T_{3}\left(x,\omega \right)}{\partial x}\right|}_{x=0}-{\left.K_{1}\frac{\partial T_{1}\left(x,\omega \right)}{\partial x}\right|}_{x=0}=-z_{th}T_{1}\left(0,\omega \right).$$

(10) $$r_{th}=l/K_{2},\;z_{th}=i\omega \rho_{2}C_{2}l,$$

where *K*_2_ and *ρ*_2_*C*_2_ are the thermal conductivity and the volumetric heat capacity of the intermediate layer, respectively, and *i* is the imaginary unit. Thin layer means here that the layer thickness is less than the thermal wave length.

Equation (10) shows that the intermediate layer is characterized only by the TR in the case of a constant heat flow. In the general case of non-stationary heat flows, the intermediate layer should be characterized by both the TR and the reactive thermal conductance. The TR and the reactive capacitive thermal conductance depend on different thermal parameters of the material. The TR *r_th_* depends only on the thermal conductivity of the intermediate layer, while the capacitive thermal conductance depends on its volumetric heat capacity *ρC_p_*. The estimates given in [57] show that neglecting the reactive thermal conductance *z_th_* is possible only in the case of *η*_2_^2^ << *η*_1_*η*_3_, where *η* = (*ρC_p_K*)^1/2^ is the thermal effusivity. Otherwise, one can just ignore the TR *r_th_*.

In experiments, the thermal parameters of the intermediate layer are unknown a priori. Usually, their values are determined from the comparison of the obtained experimental data and the corresponding theoretical results. At present, such theoretical 3D models have been developed both for the cases where the intermediate layers are located parallel to the sample surface [59,60] and perpendicular to it [58,61]. Numerical algorithms taking into account the temperature discontinuity induced by the contact TR are also being developed for calculating heat fluxes in multilayer structures [56]. Theoretical calculations of heat diffusion in a 5-layer model and in a 3-layer model with two complex thermal resistances instead of the 2nd and 4th intermediate layers showed that if the intermediate layer thickness is less than 0.15 of the thermal wave length, then the layer can be replaced by the interfacial TR with an accuracy of less than 1% [58]. This result makes it possible to reduce the number of layers required for the correct description of solder joints, which greatly simplifies the quantitative analysis of the PBD signal in the case of generation of the three-dimensional thermal waves.

The expression for the normal PBD signal normalized to the probe beam intensity has the form [62]:

(11) $$S(t)=\frac{dn}{dT}\frac{1}{\pi r^{2}}\int_{-\infty }^{0}dz\int_{-\infty }^{\infty }dx\int_{-\infty }^{\infty }dy\frac{\partial }{\partial z}T_{g}(x,y,z,t)e^{-{\left(z-z_{p}\right)}^{2}/r^{2}-{\left(x-x_{p}\right)}^{2}/r^{2}},$$

where *n* is the refractive index of air, *r* is the radius of the Gaussian probe beam, *T_g_* is the temperature of the adjacent gas media, (*x_p_*, *z_p_*) are the coordinates of the probe beam center in *x* and *z* directions, and *y* is the direction of the probe beam distribution. The coordinate system is shown in Figure 1.

When calculating the PBD signal using Equation (9), it was assumed that the air temperature at the boundary with the sample coincides with the temperature of its surface. The influence of the thermal characteristics of the sample on the PBD signal is analyzed in Ref. [62]. In Refs. [58,63], the results of the *T*_g_(*x,y,z*) calculation are generalized to the case of the presence of vertical boundaries with the interfacial TR in the sample.

## 4. Evaluation of the Thermal Properties of Joint Layers in High-Power Semiconductor Devices

The experimental investigations of the heat diffusion through joints were carried out for solar cells with radiation concentrators made on InGaP/GaAs/Ge nanoheterostructures and high-power pulse semiconductor opening switches. In the solar cells, the strong heating of the semiconductor structure takes place, and efficient heat dissipation is required to extend the active life of devices. AlN substrate is used as a heat sink in InGaP/GaAs/Ge solar cells. For this reason, the quality of the thermal contact between the Ge wafer and AlN ceramics is very important. 

### 4.1. Investigation of Solder Joint Based on Sn63/Pb37 Alloy

This section presents a detailed study of solder joints made using the active solder paste WS-483 by AIM Solder consisting of Sn63/Pb37 and Sn62/Pb36/Ag2 [64].

#### 4.1.1. Effect of Solder-Layer Inhomogeneity on the Joint Thermal Resistance

**Figure 3:**
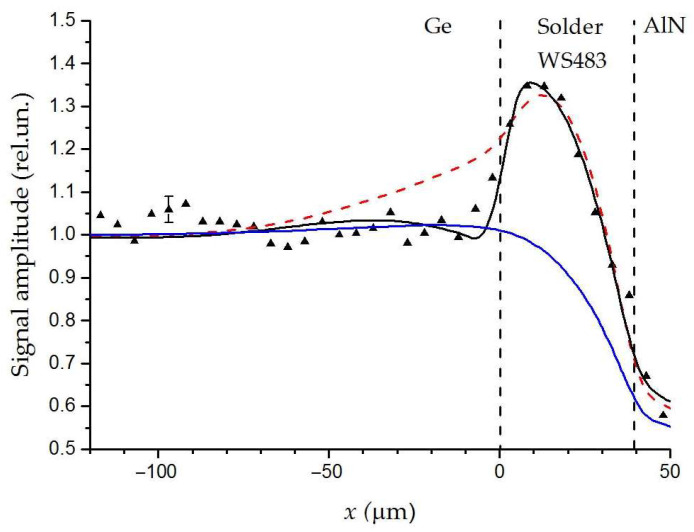
The PBD-signal amplitude. Black triangles are the experimental data. The blue curve corresponds to the solder thermal conductivity *K* = 22 W/(m K) and the perfect Ge−solder interface. The red curve corresponds to *K* = 33 W/(m K) and the Ge−solder interface with *r_th_* = 3 × 10^−6^ m^2^K/W and *z_th_* = 0. The black curve corresponds to *K* = 28 W/(m K), *r_th_* = 3 × 10^−7^ m^2^K/W, and *z_th_* = *i* 6.8 × 10^5^ Wm^−2^ K^−1^. Dash lines are solder layer boundaries. Adopted with permission from [58]. Copyright (2018), Elsevier.

During the reflow, the homogeneity of the solder composition may be disturbed due to differences in the diffusion coefficients of various types of atoms. It is revealed that solder elements can diffuse into intermediate layers under soldering and aging, forming additional alloy layers around the joint interfaces [19]. The solder joints of solar cells with an AlN substrate were experimentally studied by the PBD method to reveal factors affecting the heat diffusion. Before soldering, the surfaces of the germanium wafers and AlN ceramics are usually metallized with thin layers of silver, gold, and copper. The thickness of these layers is about a few micrometers. The solar cell components were soldered under thermal conditions and mechanical pressures recommended by the manufacturer. The average thickness of the joint layers in the resulting structures was about 40 μm [58]. Figure 3 presents the results of comparing experimental data for the PBD signal and theoretical calculations for various thermal parameters of the solder layer and boundary conditions. The unknown parameters in the model were *K* and *ρC* of the solder layer, as well as *r_th_* and *z_th_* for the interface between the Ge wafer and the solder layer. The best approximation of experimental results takes place at *K* = 28 Wm^−1^ K^−1^, *ρC* = 1.83 × 10^6^ Jm^−3^ K^−1^, *r_th_* = 3 × 10^−7^ m^2^K/W, *z_th_* = *i* 6.8 × 10^5^ Wm^−2^ K^−1^ at *f* = 2 kHz, where *r_th_* and *z_th_* refer to the interface between the solder layer and germanium. The theoretical result for the perfect contact with *r_th_* = *z_th_* = 0 and thermal parameters of the solder provided by the manufacturer (*K* = 50 Wm^−1^ K^−1^, *ρC* = 1.83 × 10^6^ Jm^−3^ K^−1^) is also presented. The results demonstrate that the variation of bulk thermal parameters of the solder layer is insufficient to ensure a good agreement with the experimental data. Even the introduction of the thermal resistance of the joint interfaces does not resolve inconsistence between the experimental and theoretical results in the case of a non-stationary heat flow. Only accounting for the reactive component of the interfacial TR makes it possible to improve the correspondence between the experimental and calculated PBD signals.

**Figure 4:**
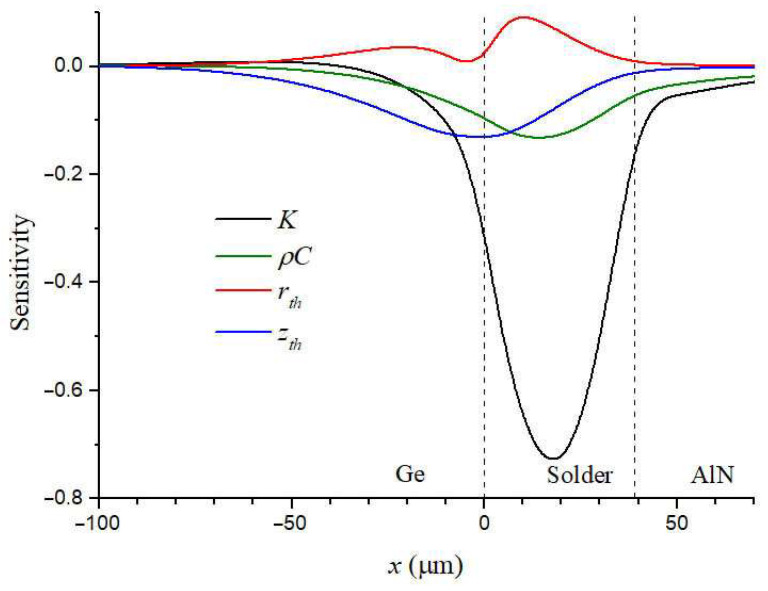
Calculated sensitivities to thermal conductivity, *K*, volumetric heat capacity, *ρC*, the interfacial TR, *r_th_*, and the reactive thermal conductance, *z_th_*, for the sample in Figure 3. Dash lines are solder layer boundaries.

The theoretical model for multilayer samples initially has a large number of unknowns. The direct determination of these by the fitting method, firstly, is very computationally expensive, and secondly, there is a high degree of probability that it will lead to an incorrect result, depending on the starting values of the parameters. This is why a preliminary sensitivity and uncertainty analysis is necessary. The algorithm is described in detail, e.g., in [65]. As a simplified example of such an analysis, let us consider the fitting procedure, the results of which are shown in Figure 4. The sensitivity of the PBD signal to a parameter *x* can be defined as ∂S/∂lnx [65].

Figure 4 shows the sensitivity to unknown thermal parameters of the joint calculated using Equation (11). It is seen that the sensitivity to *K* and sensitivity to *ρC* are closely correlated. This is because when the layer thickness is less than the thermal wave length, the temperature is mainly defined by the thermal effusivity, $\sqrt{K\rho C}$. Considering that the sensitivity to *ρC* is much less than to *K*, and the range of *ρC* for solids is narrower than the range of *K*, *ρC* was taken as a controlled parameter. The sensitivity graph shows that one can begin with fitting *K* as the first approximation. The sensitivity analysis allows reducing the region of the approximation. For example, when firstly fitting *r_th_* and *z_th_*, it can be limited to the segment [−30,30]. The obtained parameters can be used as a good starting point, which increases the reliability of the final results. The described algorithm made it possible to reduce the number of fitting parameters and the computation time.

**Figure 5:**
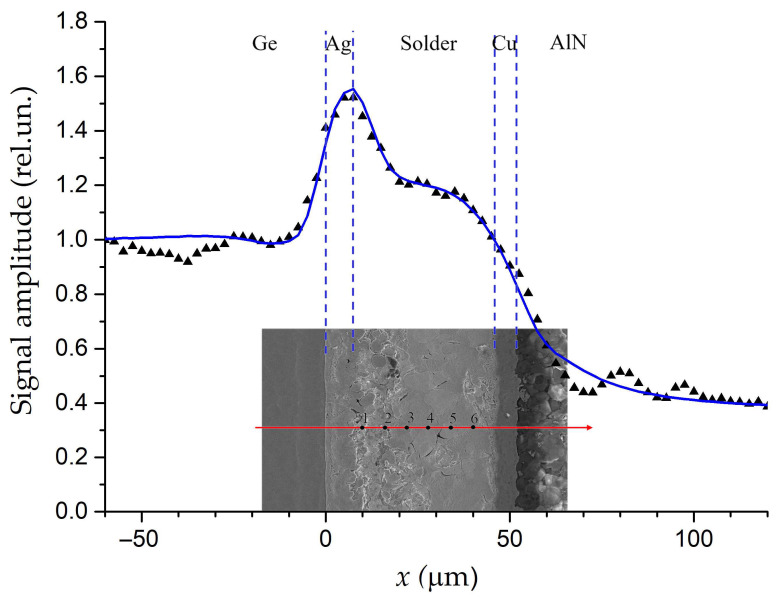
The averaged PBD-signal amplitude along the line in the SEM micrograph of area near the solder joint of the Ge−solder−AlN structure. Triangles are for experimental data. Blue curves are fitting. The element composition in the marked points on the red line is given in Table 1. Adapted with permission from [63]. Copyright (2019), Elsevier.

Moreover, the PBD images of some areas demonstrate the inhomogeneity of the solder layer, as shown in Figure 5. To elucidate the reasons of such behavior, the sample was investigated by scanning electron microscopy (SEM) and energy dispersive X-ray spectroscopy (EDS) methods [63]. These methods allow one to control the local structure and composition of the material. Figure 5 also shows the SEM image of the solder layer. Table 1 presents the composition of the layer at the checkpoints. It is seen that the solder layer structure is very inhomogeneous due to the diffusion of solder components and the partial penetration of atoms into the solder from the metallized surfaces of the Ge wafer and AlN ceramics. The silver of the contact layer on the Ge side is mixed with the bulk material. The gold that covered the copper on the AlN-ceramic side was almost completely dissolved in the solder. The almost complete penetration of the gold into the solder layer correlates well with its high solubility in Sn–Pb solders [66]. The interaction of these atoms can lead to the formation of phase boundaries [67], and it can lead to the formation of Kirkendal voids during soldering [51]. The specified inhomogeneity of the solder layer composition leads to a decrease in its effective thermal conductivity compared to the value given by the manufacturer for a homogeneous composition.

#### 4.1.2. Effect of Aging by Thermal Cycling

**Figure 6:**
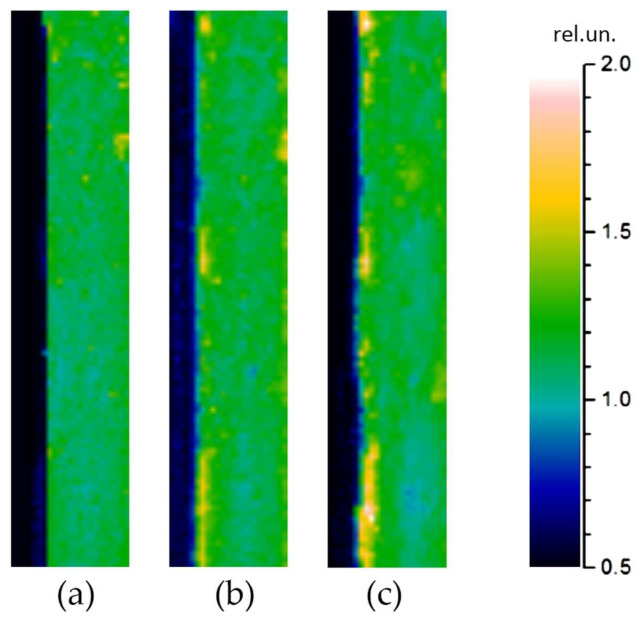
The PBD images of the area near a solder joint of Ge−Solder−AlN structure. The image size is 200 × 920 μm: (**a**) the sample in the initial state; (**b**) the sample after one cycle of cooling in liquid nitrogen; (**c**) the sample after 10 cycles. Adapted with permission from [68]. Copyright (2011), Pleiades Publishing, Inc.

An example of studying the solder joint degradation with temperature is considered in Ref. [68]. Figure 6 presents the PBD images of an area near the solder joint between the Ge wafer and AlN in the initial state (Figure 6a) after one temperature cycle (Figure 6b) and after ten temperature cycles (Figure 6c). The temperature cycle is the immersion in liquid nitrogen for 10 s followed by waiting for 1 min. White spots correspond to an increase in the signal depending on the TR. The calculated average TR was equal to 7.7 × 10^−7^ m^2^K/W for the solder layer in the initial state and increased up to 2 × 10^−6^ and 5 × 10^−6^ m^2^K/W after the 1st and the 10th immersion, respectively.

#### 4.1.3. Effect of the Surface Preparation before Soldering

**Figure 7:**
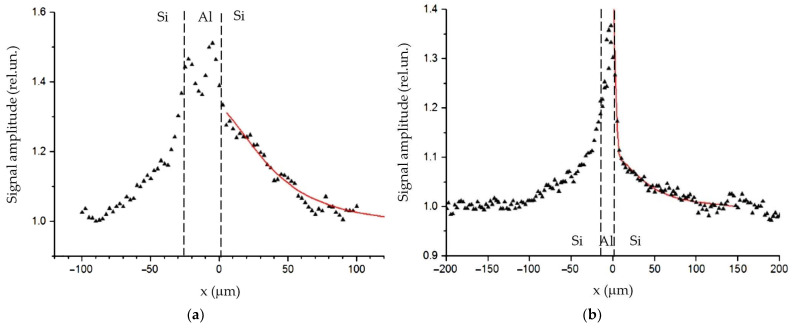
Amplitude of the PBD signal across a solder joint of two silicon diodes: (**a**) silicon wafers with ground surfaces; (**b**) silicon wafers with polished surfaces. Triangles are for experimental data. Red curves are fitting. Dash lines are joint boundaries. Adapted with permission from [70]. Copyright (2011), Pleiades Publishing, Inc.

Another important area of electronics that requires the creation of contacts with high thermal conductivity is associated with the development of high-power semiconductor devices. Among them, an important place is occupied by pulse semiconductor switches based on special diode structures [69]. Such switches are a set of coupled diode structures. Studies of the thermal properties of joints between silicon wafers soldered by paste WS-483 were presented in [70,71]. The authors investigated the joints between wafers with various quality of surfaces. Figure 7 shows the results of the PBD measurements at 1 kHz of such joints and the fitted curves. It is seen that the quality of processing strongly affects the heat-conducting properties of the contacts. The analysis of the behavior of the PBD signals shows that joints with ground surfaces are characterized by values *r_th_* = 1.9 × 10^−6^ m^2^K/W, *z_th_* = *i* 4.3 × 10^5^ Wm^−2^ K^−1^, while polished surfaces are characterized by values *r_th_* = 4.5 × 10^−7^ m^2^K/W, *z_th_* = *i* 2.0 × 10^5^ Wm^−2^ K^−1^. Such an effect of surface roughness on the thermal properties of the contact correlates with the conclusions made in [72], and it is explained by phonon scattering.

### 4.2. Investigation of the Solder Joints Based on Lead-Free Solder Pastes

**Figure 8:**
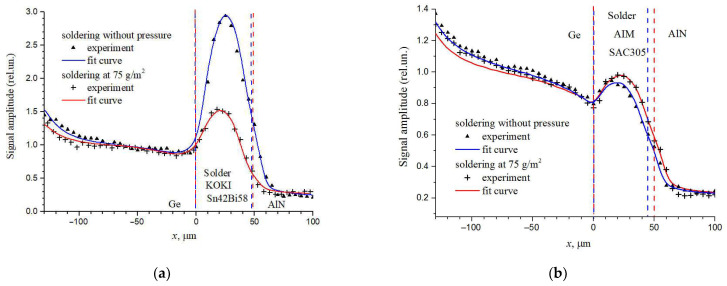
Amplitude of the PBD signal across the solder joint between the Ge wafer and the AlN ceramics: (**a**) solder KOKI; (**b**) solder AIM. Dash lines are solder layer boundaries. Adapted with permission from [75]. Copyright (2022), Ioffe Institute.

At present, serious attention is paid to the use of lead-free solder pastes. In this regard, it is important to compare their thermophysical properties with pastes containing lead. Solder joints between the Ge wafers and the AlN ceramics made from the Sn42/Bi58 binary alloy paste TB48-M742 by KOKI Company Ltd. [73] and SnAgCu0.5 ternary alloy paste REL61 M8 by AIM Solder [74] were investigated in [75]. The results of comparing the experimental and theoretical values of the PBD signals depending on the distance between the zone of the laser excitation of thermal waves and the Ge−substrate boundary are presented in Figure 8 for the solder joints made by lead-free binary KOKI and ternary alloy AIM pastes. It can be seen from the figure that the PBD signal increases as the thermal waves excitation zone approaches the solder joint from germanium, reaches the maximum inside the solder, and then decreases as it approaches the heat-removing ceramics. The figure shows that the thermal conductivity of the joint made with the paste KOKI, unlike that made with the AIM paste, increased significantly while soldered under pressure. The theoretical model of the samples consisted of four layers (glue–Ge–solder–AlN) and the complex thermal impedance at the interface between the Ge wafer and solder. The thermal conductivity of the paste AIM in both cases was about 50 Wm^−1^ K^−1^. The thermal conductivity of the paste KOKI changes noticeably from 13 Wm^−1^ K^−1^ when soldering without pressure to 26 Wm^−1^ K^−1^ when soldering under the pressure 75 g/cm^2^. It should be noted that for a good agreement between the model and the experimental data, it was necessary to add the interfacial TR for all samples. For the paste KOKI, the joint TR gradually decreases with increasing pressure from 3.7 × 10^−6^ m^2^K/W at zero pressure to 2.0 × 10^−6^ m^2^K/W at the pressure 75 g/cm^2^, while for paste AIM M8, it is almost independent of the pressure. The presence of reactive thermal conductance at the level *i* (1.8 ± 0.2) × 10^6^ Jm^−2^ K^−1^ was also added for the paste KOKI at the modulation frequency 1 kHz. The presented results show that lead-free pastes can provide the thermal conductivity at the level of lead-containing pastes. 

### 4.3. Investigation of the Bonded Joint between Silicon Wafers

**Figure 9:**
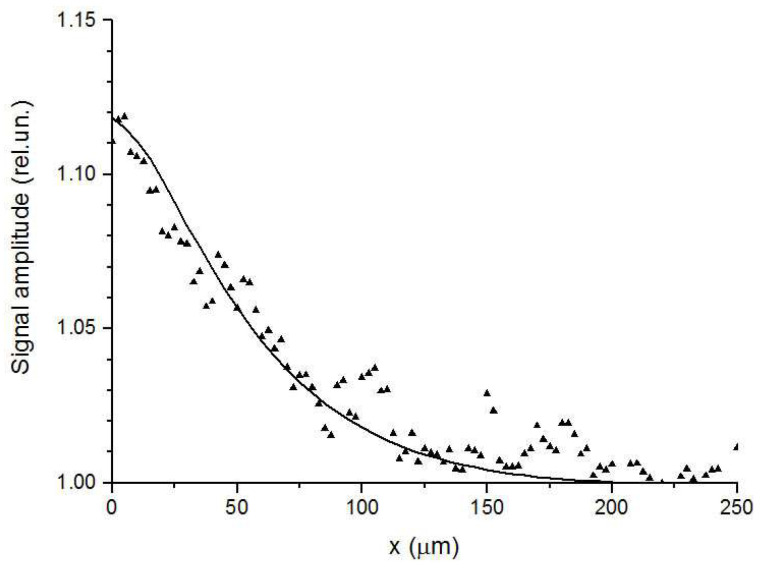
The PBD signal amplitude versus the distance from the Si–Al interface. Triangles are for the experimental data for the sample with the joint thickness of 15 μm bonded under the pressure of 5.5 kPa. The solid curve corresponds to the fitted thermal resistance 2.5 × 10^−7^ m^2^K/W. Adapted with permission from [58]. Copyright (2018), Elsevier.

Another type of joints presented in this review are made by bonding. To fabricate silicon bonded structures, aluminium layers with the thickness of several microns were deposited on the silicon surfaces of the diodes. Then, the elements were stacked and bonded together in a vacuum under pressure. Three types of samples differing in the thickness of the Al layer and the bonding pressure were investigated. The samples with joint thicknesses of 5 and 15 microns were made under the pressure of 5.5 kPa [58], and a sample with a thickness of about 30 microns was made under the pressure of 100 kPa [70]. The experimental study shows that heat transfer through the bonding joints strongly depends on the pressure. According to the fit, the TR of the 15 micron joint was equal to 2.5 × 10^−7^ m^2^K/W. The behavior of the experimental and theoretical PBD signals near the interface is shown in Figure 9. Just at the interface, the signal amplitude increased by 12%. For another sample with the joint thickness of 5 microns, the maximum signal amplitude increase did not exceed 2%. For a sample made under the pressure of 100 kPa, the signal amplitude increase near the interface did not exceed the measurement accuracy. Thus, the PBD method showed the high sensitivity in determining the TR of bonded devices. Similar sensitivity was demonstrated in the TR of bonded samples in [44], using the frequency-scan photothermal reflectance method.

## 5. Conclusions

The theoretical model and the laser PBD experimental results for joints in semiconductor structures made using various solder pastes and bonding are presented and discussed. The appearance of the interfacial thermal resistance with real and reactive components in soldered and bonded joints is demonstrated. In the theoretical model, the TR appears as a certain quantity characterizing the presence of the intermediate layer between contacting solid bodies. Such a model corresponds well to the generally accepted phenomenological approach of including the TR in the boundary conditions for temperature when solving heat problem for thermal contacts. It corresponds especially well to the case of solder joints, since according to the energy dispersive X-ray spectroscopy it takes into account the mixing of the atoms of different types near contact regions. At the same time, atoms of metals initially deposited on the contacting surfaces are also present in the composition of the real solder layers. The comparison of the laser PBD experimental and theoretical data makes it possible to obtain the quantitative thermophysical characteristics of solder joints. The data for the joints made using lead-containing solder pastes and lead-free solder pastes are presented and analyzed. It has been experimentally shown that due to the inhomogeneity of the composition of real solder joints, their thermophysical characteristics may differ from the values indicated by manufacturers. The presence of the additional interfacial TR was found for joints made using both lead-containing and lead-free pastes. The study of the soldered and bonded joints of semiconductor wafers showed that their thermal properties strongly depend on the quality of the surface treatment. The use of polished surfaces significantly reduces the interfacial thermal resistance.

The presented results demonstrate the efficiency of using the laser thermal wave techniques with the probe-beam deflection sensing for analyzing the thermophysical properties of joints. Taking into account the data presented in this review, it seems appropriate to further apply the described methodology to study the thermal parameters of a wider class of joints and contacts.

## Figures and Tables

**Table 1:** Data of the EDS analysis corresponding to Figure 5. Adopted with permission from [63]. Copyright (2019), Elsevier.

	Main Elements (wt%)			
Point	Sn	Pb	Ag	Au
1	47.41	52.59		
2	10.56	86.13	3.31	
3	24.53		75.47	
4	49.04			48.63
5	50.97			46.56
6	69.45			30.55

## Data Availability

Not applicable.
